# A patient and physician survey of fibromyalgia across Latin America and Europe

**DOI:** 10.1186/1471-2474-14-188

**Published:** 2013-06-14

**Authors:** Patricia Clark, Eduardo S Paiva, Anna Ginovker, Patricia Arline Salomón

**Affiliations:** 1 Clinical Epidemiology Unit HIM-Federico Gómez Faculty of Medicine UNAM, Colonia Doctores, DF, Mexico City, Mexico; 2 Hospital de Clinicas, Universidade Federal do Parana, Hospital de Clinicas, Curitiba, Brazil; 3 Harris Interactive, Inc., Rochester, New York, NY, USA; 4 Pfizer Inc, Paseo de los Tamarindos #40 Col. Bosques de las Lomas, DF, 05120, Mexico, Mexico

**Keywords:** Survey data, Impact of fibromyalgia, Regional comparison, Burden of disease

## Abstract

**Background:**

Patients and physicians from three Latin American (LA) and six European countries were surveyed in order to describe differences in journey to diagnosis, impact, and management of fibromyalgia (FM).

**Methods:**

900 patients (300 LA; 600 Europe) and 1824 physicians (604 LA; 1220 Europe) were surveyed between October-December 2010 (LA) and February-April 2008 (Europe). Patients and physicians (GP or specialists) completed separate questionnaires, on symptoms, impact, and FM management. Interviews were conducted in local languages. Appropriate rating scales were used throughout. Data were analyzed using cross-tabulations and descriptive statistics. Significance was determined at *P* < 0.05 (indicated by *).

**Results:**

In LA versus Europe, patients reported having FM symptoms for longer (100.8 *vs.* 83.7* months), and taking longer to be diagnosed (42.3 *vs.* 31.1* months). FM was characterized by multiple symptoms (11.2 *vs*. 6.9), but more LA patients reported 14 common symptoms*, and rated pain higher on 11-point scale (8.0 *vs*. 7.2*). LA patients were taking fewer medications (3.3 *vs*. 4.0). Patients from both regions found common symptoms very/extremely disruptive to their quality of life, but symptoms impacted daily living and ability to work more significantly in LA. Physicians (GPs or specialists) from LA more often considered problems sleeping*, difficulty concentrating*, anxiety*, depression*, numbness/tingling*, and leg cramps* very/extremely disruptive *vs*. European physicians. Despite headache, heightened sensitivity to touch, difficulty concentrating, and joint pain being experienced by ≥50% of patients from both regions, <15% of PCPs or specialists considered these typical FM symptoms. Patients also considered 12/14 symptoms more disruptive than PCPs or specialists in the same region. However, a higher proportion of PCPs or specialists considered FM to have a strong/very strong impact on aspects of daily living *vs*. patients within the same region.

**Conclusions:**

Patient- and physician-rated disease perception and impact was often higher in LA than in Europe. Patient and physician perspective concerning FM impact and disruption were often misaligned within the same region. Our observations may be representative of cultural differences in stoicism, expression, beliefs, and attitudes to pain perception and management. Better understanding of these complexities could help targeted educational/training programs incorporating cultural differences, to improve chronic care.

## Background

Fibromyalgia (FM) is a multi-factorial disease involving physiological as well as psychological factors, and characterized by widespread pain and muscle tenderness accompanied by other comorbid symptoms [1,2]. Prevalence estimates vary, with up to 5% of women reported to have FM from survey data in the US and across Europe [2-4], but some lower estimates have been presented from survey data of rheumatic diseases for other regions, such as certain Latin American countries [5-9]. Although the reasons for differences in prevalence are ultimately unknown, differences among healthcare practices, historical recognition of FM symptoms, and dissemination of guidelines on diagnosis in different regions of the world could be factors. In addition, referral bias, whereby hospital-based data produce higher estimates of disease prevalence than survey samples from the general population, also contributes to variation in the literature.

Guidelines for diagnosis and/or management of FM have been published by recognized bodies, such as the American College of Rheumatology (ACR) [1,10], and by bodies outside of the US [11-13]. Despite these protocols, FM is under-diagnosed and under-treated [14]. Physicians may diagnose patients by ruling out other conditions that share some symptoms with FM, for example chronic fatigue syndrome, rheumatoid arthritis, and multiple sclerosis [15]. Local treatment practices and diagnosis guidelines especially outside of the US and the ACR may not be widely disseminated to physicians who encounter patients with FM symptoms in their everyday practice. As a result, diagnosis and treatment can be delayed and efforts to improve recognition and diagnosis are needed [14]. For some patients, satisfaction with health status increases with a diagnosis [16], and therefore earlier diagnosis and treatment may improve treatment response and reduce the negative impact that FM symptoms have on aspects of daily living [17-20]. FM patients in some countries report frequent healthcare use and work days lost compared with both non-FM subjects and patients with other rheumatic conditions [17,21-23]. As a result of high healthcare use, studies from the US and Europe have found that FM places a significant economic burden on patients and healthcare systems [24-26]. Hidden costs of disability and comorbidities associated with FM likely increase the true burden of FM even higher.

Despite the well-reported burden FM has on activities of daily living, few data on the social and personal impact of FM on patients from Latin American countries have been published, and no data, to our knowledge, have been published examining patient and physician data from Latin America in comparison with other countries that use different healthcare practices. This international survey of FM patients and physicians from three Latin American countries and six European countries sought to examine differences in the perception and management of FM, and social and occupational aspects of FM between countries with different cultures and economies: Latin America and Europe.

## Methods

Data were collected between February-April 2008 in Europe, and October-December 2010 in Latin America. Surveyed physicians including primary care practitioners (PCPs) and specialists were identified using proprietary physician databases developed and maintained by research agencies in each of the countries, lists of professional physician associations, phone directories, and other commercially available sample sources. “*Physicians who treated FM patients*” or “*FM-treating physicians*” were defined for this survey as physicians currently seeing or who had seen at least one FM patient over the past 2 years. Patients had to have been diagnosed with FM by a physician and they were identified by physicians who treated them for FM. The recruiting physicians either participated in the survey or were sampled specifically to recruit FM patients, but did not complete the physician survey. Except for patients from Brazil, patients and physicians were compensated for completing the survey. Ethical approval for this study was not required according to regulations within the countries, at the time the surveys were carried out.

### Questionnaires

Patients and physicians completed different surveys. The sponsor had no role during the conduct of the survey, and the sponsor’s name did not appear on any of the survey materials, nor was it mentioned to patients or physicians during the survey. Therefore, the influence of the sponsor on responses by physicians or patients would be negligible. The English questionnaire was translated into each language by an independent professional translation agency, and all translations were then reviewed by a separate, independent translation agency. The trained interviewer in healthcare research who administered the questionnaire also reviewed the translations before collecting data. Patient interviews (face-to-face or via telephone) averaged 25 minutes and were conducted in local languages. Rating scales were used throughout the survey.

Questions covered symptoms (a given list of 14 common symptoms), management, and impact of FM. Physicians were also asked questions regarding their clinical background. The scales used were: *Disruption Scale* (Extremely disruptive, Very disruptive, Fairly disruptive, Not very disruptive, Not at all disruptive; top two box ratings = Extremely/Very disruptive); *Agreement Scale* (Agree strongly, Agree somewhat, Neither agree nor disagree, Disagree somewhat, Disagree strongly; top two box ratings = Agree strongly/Agree somewhat); *Impact Scale* (Very strong impact, Strong impact, Moderate impact, Slight impact, No impact; top two box ratings = Very strong/Strong impact); *Satisfaction Scale* (Extremely satisfied, Very satisfied, Satisfied, Somewhat satisfied, Not at all satisfied; top two box ratings = Extremely satisfied/Very satisfied). Pain was rated on an 11-point numerical rating scale from 0 (“No pain”) to 10 (“Worst possible pain”). Copies of the surveys can be found in the Additional file 1 and Additional file 2. All scales had the equivalent option of decline to answer or “Not known/Unsure”.

### Statistical testing

Data were processed and quality assured. Data were analyzed using cross-tabulations and descriptive statistics; no multivariate analysis was done. Significance was determined at *P* < 0.05 using *t* test of column proportions.

## Results

### Patient’s perspective of FM

The study sample included 900 patients with a confirmed diagnosis of FM according to ACR 1990 criteria [10]; 300 patients from Latin America (100 each from Mexico, Venezuela, Brazil), and 600 from Europe (100 each from UK, France, Germany, Italy, Spain, the Netherlands). The majority of patients were female (93% LA; 85% Europe) and aged 45–59 years (Table 1). Patients from Latin America reported having FM symptoms for a significantly longer time (100.8 *vs*. 83.7 months), and taking significantly longer to be diagnosed (42.3 *vs*. 31.1 months), and seeing more physicians to receive a diagnosis (5.4 *vs*. 4.0 physicians) compared with European patients, respectively (*P* < 0.05 for all). In a typical month, fewer patients from Latin America *vs*. Europe visited their physicians ≥2/month (23% *vs*. 44%; *P* < 0.05).

**Table 1:** Patient characteristics and FM symptoms reported by patients from Latin America and Europe

**Patient characteristics or symptoms (%)**	**Latin America (N = 300)**	**Europe (N = 600)**
Gender, Female	93%	85%
Age, years		
18-35	19%	22%
36-44	21%	25%
45-59	41%	33%
60-74	16%	17%
75+	1%	4%
Average number of symptoms	11.2	6.9
**Common symptoms experienced**^**a**^		
Chronic/widespread pain	92%*	62%
Problems sleeping	84%*	49%
Fatigue	88%*	46%
Headaches	84%*	61%
Facial pain	52%*	42%
Heightened sensitivity to touch	73%*	50%
Difficulty concentrating	78%*	52%
Numbness &/or tingling sensations	82%*	42%
Feelings of anxiety	79%*	28%
Feelings of depression	80%*	41%
Joint pain	89%*	59%
Stiffness	77%*	49%
Leg cramps	75%*	47%
Low back pain	83%*	55%

^a^ 14 common symptoms were given.

* *P* < 0.05.

**Figure 1:**
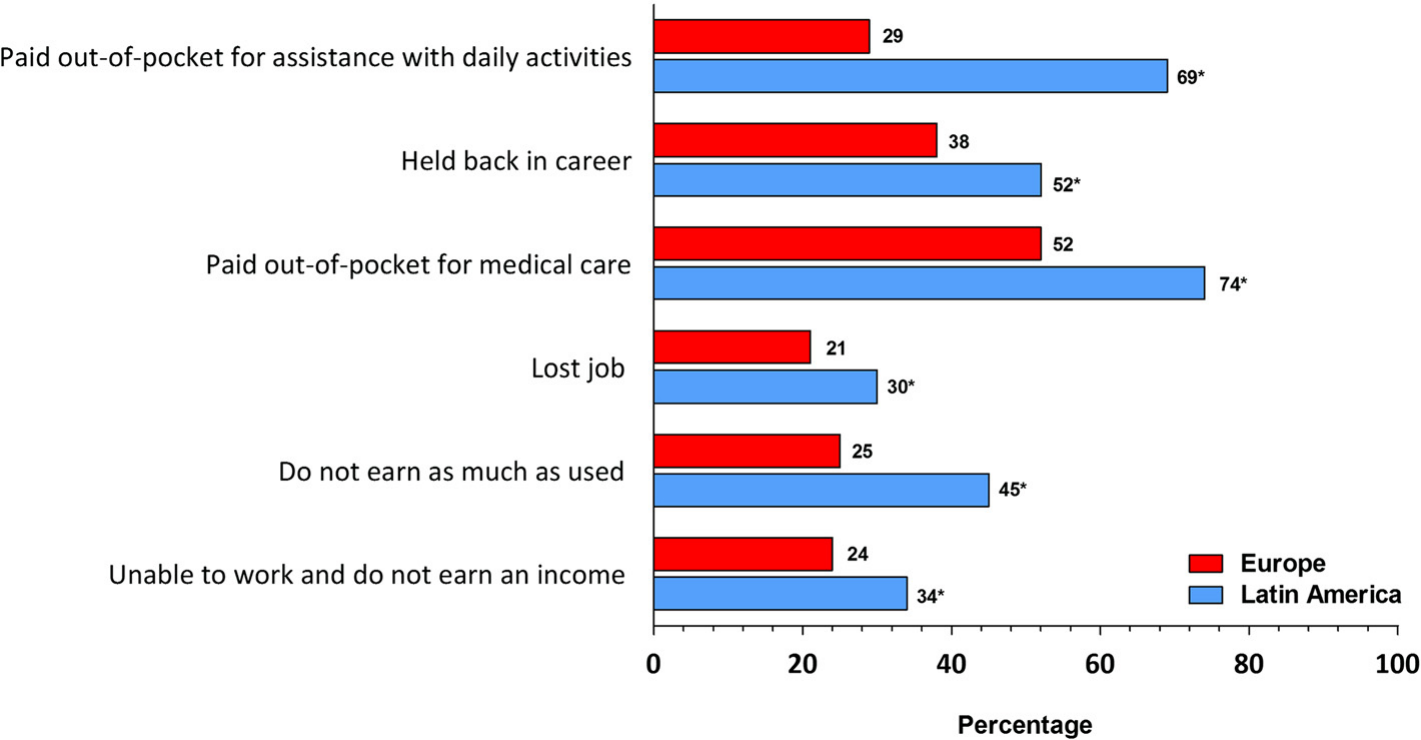
**Patient-reported impact of FM on work status and income.** * = *P* < 0.05 comparison Europe *vs*. Latin America.

**Figure 2:**
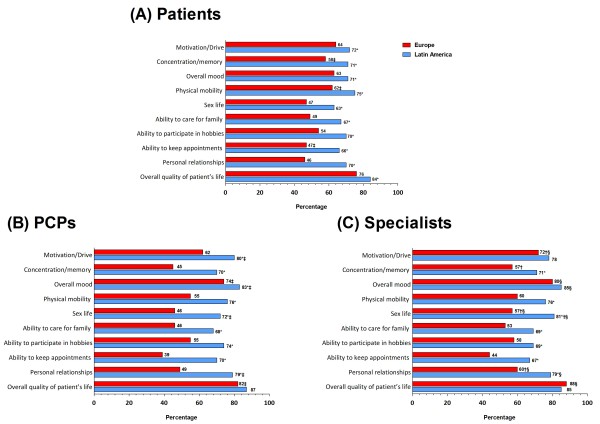
**FM has a Very Strong/Strong impact on given aspects of patient’s life, as reported by (A) patients, (B) PCPs,**^**a **^**and (C) specialists **^**a**^**. **^a^ Base case: Physicians currently seeing or having seen patients with FM; * = *P* < 0.05 Europe *vs*. Latin America within group (Patient, PCP, Specialist);^†^*P* < 0.05 PCP *vs*. specialist (within region); ‡ *P* < 0.05 patient *vs*. PCP (within region); § *P* < 0.05 patient *vs*. specialist (within region).

FM was characterized by multiple symptoms in both regions (LA: 11.2; Europe: 6.9), but a significantly higher proportion of patients from Latin America *vs*. Europe, respectively, reported common symptoms, including widespread pain (92% *vs*. 62%), sleep problems (84% *vs.* 49%), and fatigue (88% *vs.* 46%) (Table 1). Patients from Latin America also rated their pain higher on an 11-point scale *vs.* European patients (8.0 *vs.* 7.2). Patients from both regions reported common FM symptoms as disruptive (very/extremely), most commonly chronic/widespread pain (LA *vs.* Europe: 86% *vs.* 78%), sleeping problems (80% *vs.* 76%), and fatigue (80% *vs.* 75%) (Table 2). Patients from Latin America more often reported that FM impacted their ability to work and/or earn income than European patients (Figure 1). Although fewer patients from Latin America *vs*. Europe had been unemployed during the past 12-months (LA: 33%, Europe: 42%); significantly more patients from Latin America reported missing ≥40 days of work due to FM (LA 24%; Europe: 3%). Patients from Latin America also more often reported that FM had a strong or very strong impact on aspects of daily living than those from Europe, including on physical mobility, motivation/drive, and their overall quality of life (Figure 2A).

**Table 2:** Proportion of patients, PCPs, and specialists who report each symptom as “Very” or “Extremely” disruptive

	**Patients (%)**		**PCPs (%)**^**a**^		**Specialists (%) **^**a**^	
**Common symptoms**	**Latin America (N = 300)**	**Europe (N = 600)**	**Latin America (N = 253)**	**Europe (N = 503)**	**Latin America (N = 254)**	**Europe (N = 495)**
Chronic/Widespread pain	86%*‡	78%	79%	78%	80%	79%
Problems sleeping	80%‡§	76%‡§	70%*	50%	69%*	55%
Fatigue	80%‡§	75%‡§	69%	67%	66%	69%
Headaches	69%‡§	77%*‡§	52%*	37%	45%	38%
Facial pain	59%‡§	69%*‡§	40%*†	24%	30%	27%
Heightened sensitivity to touch	61%‡§	72%*‡§	50%*	42%	48%	49%†
Difficulty concentrating	73%‡§	73%‡§	51%*	36%	50%*	39%
Numbness &/or tingling sensations	59%‡§	62%‡§	48%*†	25%	37%*	24%
Feelings of anxiety	75%*‡§	64%‡§	66%*	44%	64%*	47%
Feelings of depression	73%	77%‡§	74%*	58%	73%*	62%
Joint pain	80%‡§	76%‡§	68%*†	55%	54%	53%
Stiffness	74%‡§	74%‡§	59%*†	39%	44%	39%
Leg cramps	67%*‡§	60%‡§	42%*	24%	35%*	27%
Low back pain	81%*‡§	75%‡§	59%*†	44%	50%	45%

^a^ Base case: Physicians currently seeing or have seen patients with FM; * = *P* < 0.05 Europe *vs*. Latin America within group (Patient, PCP, Specialist); † *P* < 0.05 PCP *vs*. specialist (within region); ‡ *P* < 0.05 patient *vs*. PCP (within region); § *P* < 0.05 patient *vs*. specialist (within region).

**Figure 3:**
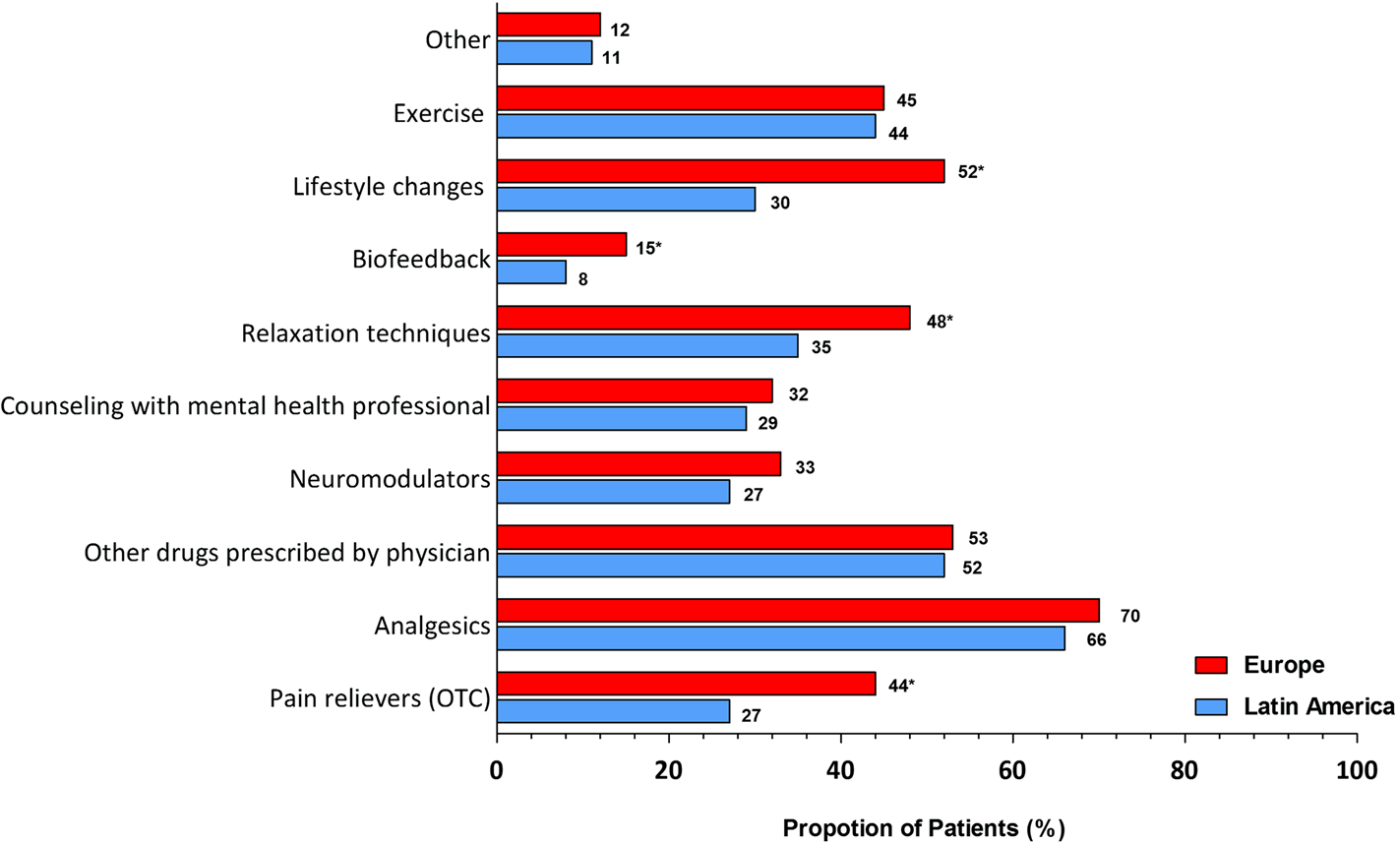
**Pharmacological and non-pharmacological treatments that patients most frequently reported receiving to treat their FM.** * = *P* < 0.05 comparison Europe *vs*. Latin America. OTC, over the counter.

On average, patients from Latin America were taking fewer medications (mean 3.3) than their European counterparts (mean 4.0). When asked what treatments they were currently using for FM, a similar proportion of patients from both regions were taking analgesics prescribed by physicians (LA: 66% *vs.* Europe: 70%), but more patients from Europe were taking over-the-counter (OTC) pain relievers (LA: 27% *vs.* Europe: 44%: *P* < 0.05) (Figure 3). Patients from Latin America were less likely than European patients to be using certain non-pharmacological therapies for their FM, including relaxation techniques, biofeedback, and lifestyle changes (Figure 3; *P* < 0.05 for all). Despite reporting use of various pharmacological and non-pharmacological therapies for FM, only 30% of Latin American patients and 20% of European patients were satisfied (extremely/very) with the ability of their treatments to relieve FM.

### Physicians’ perspective of FM

The study sample included 1824 physicians (604 LA; 1220 Europe). PCPs were general/family practitioners (LA 68%; Europe 100%) and the remainder were internists (LA 32%). Specialists were neurologists (LA: 29%; Europe: 25%), psychiatrists (LA: 26%; Europe: 28%), rheumatologists (LA: 27%; Europe: 26%), pain specialists/anaesthetists (LA: 16%; Europe 13%) and internists (LA: 1%; Europe 2%). The majority of physicians had been in practice for 11–30 years across regions, and were treating ≤20 FM patients, regardless of speciality (Table 3). PCPs saw more patients per month in each region than specialists, but, as would be expected, specialists in each region saw more FM patients than PCP. The majority of physicians were aware of 1990 ACR criteria, although the proportion of PCPs and specialists aware of and working in practices that employed these criteria was greater in Latin America than in Europe. Of 14 common symptoms, physicians similarly considered chronic/widespread pain a typical symptom in Latin America and in Europe, for PCPs (79% *vs.* 78%) and specialists (80% *vs.* 79%), respectively. Both sets of physicians from Latin America more often considered problems sleeping, difficulty concentrating, anxiety, depression, numbness/tingling, and leg cramps as disruptive (very/extremely) *vs.* European physicians (Table 2). More than 82% of PCPs and specialists from both regions indicated that FM has a very strong or strong impact on patients overall quality of life (Figure 2B and C). More PCPs from Latin America than from Europe, however, considered FM to have a very strong or strong impact on the individual aspects of daily living (Figure 2B). Generally, similar proportions of specialists from Latin America *vs*. Europe rated impact on aspects of daily living (Figure 2C). When compared within Europe, differences were recorded between PCPs and specialists responses on the impact that FM has aspects of daily living. Within Latin America the responses from PCPs and specialists were similar for all questions of daily living, except for the impact of FM on sex life (Figure 2B and C).

**Table 3:** Selected physician characteristics

	**Europe**		**Latin America**	
	**PCP**	**Specialist**	**PCP**	**Specialist**
	**N = 609**	**N = 611**	**N = 306**	**N = 298**
**Years in practice**	21.2*	19.7	16.5	18.4*
1–10 years	103 (16.9%)	136 (22.3%)	123 (40.2%)	90 (30.2%)
11–30 years	434 (71.3%)	384 (62.8%)	155 (50.7%)	161 (54.0%)
≥31 years	71† (11.7%)	91 (14.9%)	28 (9.2%)	43† (14.4%)
**How many patients do you see per month**	473.1*	250.3	271.5*	191.8
≤100	60 (9.9%)	171 (28.0%)	77 (25.2%)	121 (40.6%)
101-300	153 (25.1%)	275 (45.0%)	144 (47.1%)	112 (37.6%)
≥300	392† (65.0%)	163† (26.7%)	81† (26.5%)	60† (20.1%)
**Have seen FM patients in past 2 years (including currently)**	n = 503	n = 495	n = 253	n = 254
**How many FM patients have you seen in the past 2 years (including currently)**^**a**^	20.0	61.0*	53.9	145.7*
1-20	401 (79.7%)	270 (54.5%)	169 (66.8%)	140 (55.1%)
21-100	72 (14.3%)	150 (30.3%)	57 (22.5%)	71 (28.0%)
≥101	28† (5.6%)	73† (14.7%)	26† (10.3%)	42† (16.5%)
**Are you aware of ACR 1990 criteria**				
Yes	249 (40.9%)	341 (55.8%)	221 (72.2%)	227 (76.2%)
**Does your practice use ACR 1990 criteria to diagnose FM patients**				
Yes	151 (24.8%)	218 (35.7%)	165 (53.9%)	184 (61.7%)

* significant difference PCP *vs*. specialists within region.

^a^ use base case = currently seeing/have seen FM patients in past 2 years.

† remainder declined to answer.

### Examining patient and physician perspectives

Chronic/widespread pain was a common symptom experienced by the majority of patients, and was considered a typical FM symptom by the majority of physicians (PCPs, and specialists, respectively) from both regions. But, despite headache, heightened sensitivity to touch, difficulty concentrating, and joint pain being experienced by ≥50% of patients from both regions (Table 1), <15% of PCPs or specialists considered these typical FM symptoms (Table 4).

**Table 4:** Typical symptoms that physicians look for when diagnosing FM

	**Europe**		**Latin America**	
**Symptom **^**†**^	**PCP**	**Specialist**	**PCP**	**Specialist**
	**N = 503**	**N = 495**	**N = 253**	**N = 254**
**Chronic/widespread pain**	55%	54%	68%	74%
**Problems sleeping**	1%	2%	4%	2%
**Fatigue**	7%	9%	5%	4%
**Headaches**	0	0	0	1%
**Facial pain**	0	1%	2%	0
**Heightened sensitivity to touch**	14%	14%	7%	5%
**Difficulty concentrating**	1%	1%	0	0
**Numbness &/or tingling sensations**	3%	2%	2%	1%
**Feelings of anxiety**	0	1%	0	0
**Feelings of depression**	4%	4%	2%	1%
**Joint pain**	7%	5%	3%	3%
**Stiffness**	3%	2%	2%	2%
**Leg cramps**	0	1%	1%	0
**Low back pain**	3%	1%	3%	3%

^**†**^ 14 common symptoms were given. Base case = physicians currently seeing or having seen patients with FM.

Chronic/widespread pain was considered disruptive (very/extremely) by ≥78% of patients, PCPs and specialists from both regions. Opinions on the disruption caused by the majority of the other 14 listed FM symptoms were not aligned among patients and PCPs or specialists within the same region. For example, significantly more patients within Latin America and within Europe considered at least 12/14 symptoms more disruptive than PCPs or specialists within the same region (Table 2). The proportion of patients and physicians (PCPs or specialists) within the same region also differed on the impact caused by certain FM symptoms on aspects of daily living but, interestingly, a higher proportion of PCPs from Latin America, or specialists from both regions, considered FM had a strong or very strong impact on aspects of daily living compared with patients within the same region (Figure 2).

## Discussion

A wealth of data exists on the burden of FM from individual countries, particularly from the United States. However data from within the Latin American region and healthcare setting is particularly sparse [27] and no study has described the Latin American perspective of FM alongside patients from a different region. This study describes how FM is perceived and managed across different healthcare settings using two regions with different cultures and economies: Latin America and Europe. Consistent with reports from other countries [17,22,28], FM is reported by patients and physicians to be a debilitating chronic/widespread pain condition, more common in women, and which is characterized by multiple symptoms that negatively impact daily living. The negative impact FM had on patients and the disruption symptoms caused to aspects of daily living are also broadly in line with survey data from other regions [17,19,20,22], and reinforce the high burden FM imparts on patients, regardless of region of origin. Given the range of the data surveyed, some divergence would be expected. Nevertheless, our study presents many new observations in these populations, in addition to highlighting important differences, particularly between the impact and disruption caused by FM from a physician and patient viewpoint, within the same region. Our data therefore highlight some misalignment in perceptions of FM that need to be addressed and warrant further investigation.

Our analysis extends individual reports from within Latin America or Europe [5,7,19,29] to describe differences between patient and physician experiences regarding how FM is diagnosed, perceived, and managed. For example, patients from Latin America reported having FM symptoms for significantly longer time, and taking significantly longer to be diagnosed, and seeing more physicians to receive a diagnosis compared with European patients. Studies from the United States indicate that patients often go 5 years before receiving a correct diagnosis of FM [14,17,30], suggesting that despite the differences in journey to diagnosis patients from both regions herein were diagnosed relatively quickly (LA: 3.5 years; Europe: 2.5 years). The time difference seen between regions is representative of the work-up patients with FM face when a diagnosis is unclear, particularly historically when FM was less well defined and understood. For example, pain or other symptoms (eg., fatigue, sleep problems, depression, etc.) may have been treated by physicians as separate conditions, or even disregarded if they considered there to be a lack of medical evidence for the pain [31]. These and similar issues are inherent variables some patients face prior to receiving a confirmed diagnosis of FM [14]. Physicians, both PCPs and specialists, were generally aware of the ACR 1990 diagnosis guidelines; however they were more widely known and adopted in Latin America vs. European practices. This could be representative of European practices adopting European diagnosis or management practices that are more locally disseminated to PCPs and specialists [32]. Evidence suggests that receiving an accurate diagnosis is the first step to effective care and better outcomes for patients [14]. As such, differences in time to diagnosis in Latin America compared with Europe may have influenced disease characteristics. For example, a higher proportion of patients from Latin America reported common symptoms, and reported symptoms as being disruptive, than their European counterparts. Other factors influencing journey to diagnosis and FM experience between regions should also be considered, including patients being considered in different countries the responsibility of primary care vs. rheumatology or other specialist, which will in turn influence treatment practices as discussed in detail below.

Data were collected across different countries and healthcare systems that have different treatment practices and cultures and some of the differences observed in the FM experience may be reflective of these cultures and practices. For example, although some data have reported that clinical characteristics and patient perceptions are broadly similar across different regions [17,33,34], important ethnic, racial, and sociocultural differences between populations in pain perception have been noted [35-38]. Given the ethnic diversity of populations surveyed, these factors may well have influenced responses provided, and account for some of the differences in disease experiences recorded, although the magnitude of these influences remains unknown. Zborowski and Zola published pioneering work investigating ethnic and cultural differences in the experience of pain, including attitudes and behavioral aspects that are generated after pain, the differences in impact of the disease, and what patients from different ethnic backgrounds do in order to find help or a cure for their pain [39,40]. Since this early work, numerous authors have published cultural differences regarding stoicism, expression, beliefs, and attitudes with regard to pain and pain perception. The ways in which patients and healthcare providers interact is also influenced by ethnicity, particularly when a painful condition is present [41], and in certain ethnic groups pain is reportedly undertreated [42]. For many questions in the present study, patients from Latin America rated responses significantly higher than in Europe, which may be representative of the more “expressive” nature of patients from Latin American and the more stoic European population [41]. How these differences in journey to diagnosis and expression of FM may ultimately impact FM prevalence, which is generally lower in Latin American countries compared with Europe [3,5,9,29,43], would need further study. As such, the generalizability of the data to other countries or other FM patient populations cannot be assumed.

Patients from both regions reported using pharmacological and non-pharmacological approaches to manage their FM symptoms, and differences noted between regions provide some important insights into healthcare practices between Europe and Latin America. For example, aerobic exercise is helpful for some FM patients [44,45] and it is somewhat surprising that less than 50% of patients reported exercise therapy in either region. This may suggest that when appropriate, physicians should encourage their patients to participate in some form of exercise. An interesting area of further study in these regions would be to categorize the type and/or schedule of exercise, e.g., giving subtypes of exercise (gentle, aerobic, walking, etc.) may change the proportion of patients responding. For example, a study comparing FM management in Germany *vs*. the US reported that “aerobic exercise” was used by 32% of the US consumers compared with 58% German consumers; however gentle exercise was much higher (64% *vs* 80%) [46]. Other non-pharmacological treatment options, such as relaxation techniques, biofeedback, and lifestyle changes, were more commonly used in Europe. Given differences in economy for some Latin American *vs*. European countries, these treatments may not be easily accessible and/or reimbursed, and their utility and effectiveness is therefore harder to demonstrate in Latin America. From a pharmacological perspective, FM was managed by multiple medications, albeit fewer in Latin America than in Europe, which as noted above, may have influenced number and negative impact of FM symptoms in Latin America *vs*. Europe. Physician treatment practices have been shown to vary depending on ethnicity [42,47]. These inherent variables may have influenced prescribing practices in Latin America *vs.* Europe, especially considering fewer symptoms were experienced by patients in Europe. OTC medications were used by up to 70% of patients in both regions, highlighting that patients may have been self-managing their symptoms, in addition to, or instead of their prescriptions. Although the specific OTC therapies used were not captured and will have varied among countries surveyed even within-region, over half of all patients reported out-of-pocket costs in both regions, albeit significantly more in patients from Latin America than Europe. Although not quantified by actual costs, the wider economic impact of FM was most notable in Latin America, with almost half of employed patients missing more than 40 days’ work in the past year. Efforts should be made to help keep patients working, as long-time sick leave has been found detrimental to long-term recovery [48,49]. As a result, energy conservation, occupational therapy, evaluation, and work adaptation are important aspects of FM management [49,50] and our survey suggests in particular that physicians in Latin America should be made aware and help keep patients in work.

We report that many different types of physicians are seeing FM patients in these two healthcare settings, probably due to the diverse range of symptoms that are involved in the condition. Our study highlights that in Europe more patients were seen in primary care by a general practitioner, compared with 32% seen by internists for primary care in Latin America. A range of specialists were seeing FM patients in both regions. The ability to conduct an efficient and accurate assessment and diagnosis will depend on the physician’s background knowledge and training [51,52], and the differences in training experiences between countries may have influenced patients’ journey to diagnosis, as discussed previously. For example, ACR diagnosis criteria are used in many countries outside of the United States, including most often in Latin American countries and across some parts of Europe. Regional knowledge of diagnosis guidelines will vary, however. Recent advances in the understanding of the pathophysiology of FM may not have extended to non-specialists in primary care, and may also have differed between regions as indicated by the differences in knowledge and adoption of the ACR criteria, for example. In addition, updated ACR diagnosis criteria [1] and other guidelines outside of the US [12,32] reflect that generalized pain does not adequately characterize FM and a broader assessment of pain, function, and psychosocial aspects may aid in FM diagnosis and, ultimately, management. These observations are supported by the symptoms experienced by patients from both regions in the present survey. Updated diagnosis guidelines were released by ACR in May 2010, just prior to the Latin American survey. As a result, these updated guidelines may have increased general awareness of FM. Indeed, although pain was the principal symptom in both regions, Latin American physicians more often considered a wider range of typical FM symptoms as “Very” or “Extremely” disruptive to their patients than their European counterparts, many of which are consistent with the updated ACR diagnosis guidelines. With growing acceptance and understanding of FM, there seems a need to include focused education for physicians regarding FM, starting ideally at medical school, to ensure all types of physician have a basic understanding of the condition when encountered. It is also paramount there is a better understanding, particularly in non-specialists, of mechanisms of chronic *vs*. acute pain in order to fully understand the physiology of FM and other chronic pain conditions. This is particularly important given the different approaches to management required for pain of different origins, in addition to effective management of comorbidities and the multi-factorial symptoms of FM [53]. Enhancing educational initiatives in both countries may help improve recognition, diagnosis, and patient satisfaction with management in the longer term [54-56]. We also suggest that further efforts to implement structured treatment paradigms, incorporating pharmacological and non-pharmacological approaches for FM, may likewise guide physicians and improve confidence in treatment choices most appropriate for FM.

It is well known that a range of physicians encounter patients with FM; however, few studies have commented on how patients and physicians perceive FM differently and furthermore how this is influenced by physician type. Overall, FM symptoms were reported by patients and physicians from both regions to be “Very” or “Extremely” disruptive, and having a negative impact on many aspects of daily living. More physicians (PCPs or specialists) from Latin America considered recognized somatic symptoms of FM (per [1,10]), such as problems sleeping, difficulty concentrating, depression, and numbness/tingling, as disruptive (Very/Extremely) than their European counterparts. Nevertheless, more Latin American patients rated FM symptoms as disruptive than their physicians. This suggests that although physicians in Latin America are more aware of the disruption caused by common FM symptoms than their European counterparts, their perceptions still fall short of patients from the same region. Despite these differences in opinions on disruption of FM symptoms, interestingly within Latin America, more physicians (PCPs or specialists) rated that FM had a “Very strong” or “Strong” impact on mood, sex life, and personal relationships than patients themselves, and furthermore matched patient’s responses to all other aspects of daily living. Within Europe, more specialists likewise rated that FM had a very strong or strong impact on some aspects of daily living than European patients themselves, although responses from patients and PCPs in Europe were generally better aligned. Our observations suggest effort is needed to align patient’s and physician’s perception of FM within these regions. For example, quality of life questionnaires alongside educational initiatives could help draw attention to which FM symptoms are most disruptive and important to the patient. These types of questionnaires are not widely used in private practice, despite being translated into different languages [57-60]. Collectively, our observations indicate that there is still a long way to go concerning optimizing management of FM, irrespective of ethnic background and prescribing practices.

Our study should be interpreted in light of some limitations. The World Health Organization (WHO) recognized and classified fibromyalgia in 1993, yet in some countries doctors will have been unfamiliar with the condition, diagnosis criteria and/or treatment practices. In some countries included in our survey, no treatments are specifically approved for the treatment of FM and therefore management of patients both with prescription products and off label where no products are specifically available may have differed, particularly to countries where clear treatment protocols for FM are accepted and recommended. We sought to ensure that the diagnosis of FM was not entirely dependent on the patient’s recollection, by only including patients who were diagnosed with FM by a physician according to standard criteria [10]. FM is a clinical diagnosis and previous research suggests physicians express difficulties in diagnosis and managing FM, and confidence varied depending on specialty [51]. Therefore whether or not physicians were board-certified, and also if physicians had experience of FM sufficient to make a differential diagnosis, are inherent variables for some of the questions posed in the survey. We therefore ensured that medical evidence was available for FM being present, to limit the possibility of physicians disregarding the pain condition due to lack of medical evidence [31]. An important limitation of all opinion research, to which this study is not an exception, is that respondents may not perfectly recall their experiences and feelings at the time of the survey, as respondents' feelings, attitudes, and perceptions are subject to change with time. The survey only provides a snapshot of the respondents' experiences and does not seek to address how these might have changed longitudinally. Therefore, the observations should be considered within the time-frame they were captured, which differed slightly between regions as noted above. Although the numbers of respondents per country were the same, data were collected from more European countries than Latin American countries, increasing the diversity of responders within the European dataset. Despite these limitations, our study provides new insights into the perceptions and management of FM patients in countries outside of the US, and identified some potential areas for targeted improvement.

## Conclusions

Differences between FM characteristics, treatment practices, and opinions were noted by physicians and patients from Latin America and Europe and interestingly patient- and physician-rated disease impact and perception was often higher in Latin America than in Europe. Improved understanding of these complexities involved in FM in different healthcare settings may help target educational/training programs towards improving aspects of chronic care. As patient’s and physician’s perspectives concerning FM impact and disruption were often misaligned within the same region, there is a clear need to focus on understanding and ultimately improving these conflicting views in order to optimize chronic care.

## Competing interests

PATRICIA CLARK, MD, PHD: In the past years, Dr. Clark has received consulting fees from Pfizer related to epidemiology work Dr. Clark is not involved in drug research or consultancy; Dr Clark does not hold stocks from any company and is not applying for any patents at the time of submission.

EDUARDO S. PAIVA, MD: In the past five years, Dr Paiva has received fees for lectures in medications used in the treatment of fibromyalgia, from Pfizer Inc and Lilly, and has received fees from Pfizer Inc to present findings of this survey to journalists. Dr Paiva does not holds stocks from any company and is not applying for any patents at the time of submission.

ANNA GINOVKER, PHD: of Harris Interactive was a paid consultant to Pfizer Inc in connection with the conduct of this study.

PATRICIA ARLINE SALOMÓN, MD: is an employee of Pfizer Inc and hold stock options from Pfizer Inc.

## Authors’ contributions

AG was involved in the conception and design of the survey. All authors were involved in either the acquisition or analysis and interpretation of the data. All authors were involved in drafting the manuscript and revising it critically during development. All authors read and approved the final draft of the manuscript.

## Authors’ information

PC is head of the Clinical Epidemiology Unit, and the Hospital Infantil Federico Gómez-faculty of Medicne UNAM; and an International Fellow at the American College of Rheumatology. ESP is an Assistant Professor at the Universidade Federal do Parana, Curitiba, Brazil; Chief, Fibromyalgia Clinic, Universidade Federal do Parana, Curitiba, Brazil; and an International Fellow at the American College of Rheumatology.

## Pre-publication history

The pre-publication history for this paper can be accessed here:

http://www.biomedcentral.com/1471-2474/14/188/prepub

## Supplementary Material

Additional file 1 Physician questionnaire. Click here for file

Additional file 2 **Patient questionnaire **^**a**^**. **^a^ Published previously by: Choy E, Perrot S, Leon T, Kaplan J, Petersel D, Ginovker A, Kramer E: A patient survey of the impact of fibromyalgia and the journey to diagnosis. *BMC Health Serv Res* 2010, 10:102 [17]. Click here for file
