# Supplementary material for: A patient and physician survey of fibromyalgia across Latin America and Europe
Source: BMC Musculoskelet Disord. 2013 Jun 14;14:188. doi: 10.1186/1471-2474-14-188 (PMC3689629; doi:10.1186/1471-2474-14-188)
Supplement: Additional file 1 — Physician questionnaire. [file 1471-2474-14-188-S1.pdf]

HARRIS INTERACTIVE
5 Independence Way
Princeton, NJ 08540

Researcher: Anna Ginovker
Email: aginovker@harrisinteractive.com
Phone: 609-919-2638

Kelly Wolfe
Email: kwolfe@harrisinteractive.com
Phone: 424-263-2145

Project Manager: Michi Chan
Email: mchan@harrisinteractive.com
Phone: 585-214-7961

J38889B

Fibromyalgia in Latin America – Patient Survey

CLIENT: Pfizer

Information for Online Surveys

Title for initial survey page (Required for online jobs): Health Survey

Wave Number/Version/Languages (if applicable): Mexican Spanish, Brazilian Portuguese, Venezuelan Spanish

HARRIS INTERACTIVE

Published previously by: Choy E, et al BMC Health Serv Res 2010, 10:102 [14]

SECTION 400: PRELOADS/SCREENING QUESTIONS

BASE: ALL RESPONDENTS
Q455 (QV10) COUNTRY

1. MEXICO
2. BRAZIL
3. VENEZUELA

BASE: ALL RESPONDENTS
Q404

Thank you very much for agreeing to participate in our research! We invite you to participate in this survey about pain to tell us about some of your experiences and opinions. Your participation will help make this important study a success.

BASE: ALL RESPONDENTS
Q900 FOR INTERVIEWER USE ONLY (DO NOT READ): Please indicate below how the interview is/was administered.

1 - Administered using online survey program
2 - Administered using pen and paper questionnaire

BASE: ALL RESPONDENTS
Q410 Which of the following categories best describes your age?
(INTERVIEWER: IF RESPONDENT IS NOT SURE OR DECLINES TO ANSWER, PROBE TO CONFIRM RESPONDENT IS AT LEAST 18 YEARS OF AGE OR OLDER. GENTLY PROBE FOR APPROXIMATE AGE RANGE. UPDATE RESPONSE CHOICE AS NECESSARY. RESPONDENT MUST CONTINUE EVEN IF THE AGE RANGE IS NOT IDENTIFIED UNLESS THERE IS A STRONG ENOUGH REASON TO BELIEVE THAT RESPONDENT IS UNDER THE AGE OF 18.)

1. Under 18 [TERMINATE AND SKIP TO Q480]
2. 18-35
3. 36-44
4. 45-59
5. 60-74
6. 75 and over
8. Not sure (V)
9. Decline to answer (V)

BASE: ALL RESPONDENTS AGED 18+ (Q410/2-9)
Q412 GENDER (INTERVIEWER: RECORD GENDER. DO NOT ASK)

1. MALE
2. FEMALE

HARRIS INTERACTIVE

Published previously by: Choy E, et al BMC Health Serv Res 2010, 10:102 [14]

BASE: MEXICO, BRAZIL, VENEZUELA AND 18+ (Q455/1-3 AND Q410/2-9)
Q435 **HAS A PHYSICIAN DIAGNOSED YOU WITH FIBROMYALGIA?**

1. Yes
2. No TERMINATE
8. Not sure (V) TERMINATE
9. Decline to answer (V) TERMINATE

BASE: ALL RESPONDENTS
Q480 – INITIAL CLASSIFICATION QUESTION – BEHIND THE SCENES

PROGRAMMER NOTE:
GET CODE 1 IF:

- 18 years or older, OR Not sure, OR Decline to answer (Q410/2-9) AND
- Reside in Mexico, Brazil or Venezuela (Q455/1-3) AND
- Diagnosed with fibromyalgia (Q435/1)

ALL OTHERS GET CODE 2

1. Qualified
2. Not Qualified

HARRIS INTERACTIVE

Published previously by: Choy E, et al BMC Health Serv Res 2010, 10:102 [14]

SECTION 500: MAIN QUESTIONNAIRE—FIBROMYALGIA SYMPTOMS

Now, I am going to ask you some questions about your fibromyalgia symptoms.

BASE: ALL QUALIFIED RESPONDENTS (Q99/1)
Q500 Please indicate which of the following symptoms of fibromyalgia you have experienced. (INTERVIEWER READ LIST. ALLOW MULTIPLE RESPONSES.)

[RANDOMIZE IF Q900/1]

1. Chronic widespread pain
2. Problems sleeping
3. Fatigue
4. Headaches
5. Facial pain
6. Heightened sensitivity to touch
7. Difficulty concentrating
8. Numbness and/or tingling sensations
9. Feelings of anxiety
10. Feelings of depression
11. Joint pain
12. Stiffness
13. Leg cramps
14. Low back pain
96. Other [ANCHOR]
97. None of these [ANCHOR, E]
98. Not sure (V,) (Exclusive)
99. Decline to answer (V,) Exclusive

HARRIS INTERACTIVE

Published previously by: Choy E, et al BMC Health Serv Res 2010, 10:102 [14]

BASE: QUALIFIED RESPONDENTS WHO MENTIONED AT LEAST ONE LISTED SYMPTOM (Q500/1-14,96)

Q510 Please tell me how disruptive each of these symptoms is to the overall quality of your life. Would you say it is not at all disruptive, not very disruptive, fairly disruptive, very disruptive, or extremely disruptive? (INTERVIEWER: READ LIST AND REPEAT SCALE IF NECESSARY.)

[PROGRAMMER: DISPLAY ONLY SYMPTOMS THAT RESPONDENT MENTIONED IN Q500.]

Q511

1	2	3	4	5	8	9
Not at all disruptive	Not very disruptive	Fairly disruptive	Very disruptive	Extremely disruptive	Not Sure (V)	Decline to Answer (V)

[RANDOMIZE IF Q900/1]

1. Chronic widespread pain
2. Problems sleeping
3. Fatigue
4. Headaches
5. Facial pain
6. Heightened sensitivity to touch
7. Difficulty concentrating
8. Numbness and/or tingling sensations
9. Feelings of anxiety
10. Feelings of depression
11. Joint pain
12. Stiffness
13. Leg cramps
14. Low back pain
96. Other

BASE: RESPONDENTS WHO HAVE EXPERIENCED CHRONIC WIDESPREAD PAIN (Q500/1)

Q515 In a typical week, how often would you say you experience chronic widespread pain from fibromyalgia? Would you say…? (INTERVIEWER: READ LIST)

1. Less than once a week
2. Once a week
3. 2 to 3 times per week
4. 4 to 5 times per week
5. Every day
8. Not sure (V)
9. Decline to answer (V)

HARRIS INTERACTIVE

Published previously by: Choy E, et al BMC Health Serv Res 2010, 10:102 [14]

BASE: RESPONDENTS WHO HAVE EXPERIENCED CHRONIC WIDESPREAD PAIN (Q500/1)

Q520 In general, how severe is the chronic widespread pain you experience from your fibromyalgia? Please use a scale from 0-10, where '0' means no pain and '10' means the worst pain imaginable.

1. 0 – No pain
2. 1
3. 2
4. 3
5. 4
6. 5
7. 6
8. 7
9. 8
10. 9
11. 10 – Worst pain

98 Not sure (V)
99 Decline to answer (V)

BASE: ALL QUALIFIED RESPONDENTS (Q99/1)

Q525 How long ago did you first notice the symptoms of fibromyalgia?

(INTERVIEWER: RECORD LENGTH OF TIME AS INDICATED BY RESPONDENT.

- IF RESPONDENT PROVIDES RESPONSE THAT IS LESS THAN ONE MONTH, I.E 3 WEEKS, RECORD AS ONE MONTH.)

I_I_I year(s) (Example: 03)

Q526

I_I_I month(s) (Example: 10)
[YEARS AND MONTHS MANDATORY (DOUBLE DIGIT)]
[RANGE: 0-95]

Q527

98 – Not Sure (V) Exclusive
99 – Decline to Answer (V) Exclusive

BASE: ALL QUALIFIED RESPONDENTS (Q99/1)
Q529 BEHIND THE SCENES CALCULATION

[PROGRAMMER: CALCULATE TOTAL TIME IN MONTHS BASED ON 1 YEAR = 12 MONTHS (X YEARS*12) + MONTHS INDICATED FROM Q525]

L_L_L MONTH(S)

HARRIS INTERACTIVE

Published previously by: Choy E, et al BMC Health Serv Res 2010, 10:102 [14]

BASE: ALL QUALIFIED RESPONDENTS (Q99/1)
Q530 How long did it take you to see a physician after you first experienced your fibromyalgia symptoms?

(INTERVIEWER: RECORD LENGTH OF TIME AS INDICATED BY RESPONDENT.

- IF RESPONDENT PROVIDES RESPONSE THAT IS LESS THAN ONE WEEK, I.E 5 DAYS, RECORD AS ONE WEEK.)

I_I_I year(s) (Example: 03)

Q531 I_I_I month(s) (Example: 10)

Q532 I_I_I week(s) (Example: 01)
[YEARS, MONTHS, AND WEEKS MANDATORY (DOUBLE DIGIT)]
[RANGE: 00-95]

Q533 98 Not Sure (V) Exclusive
99 Decline to Answer (V) Exclusive

BASE: ALL QUALIFIED RESPONDENTS (Q99/1)
Q553 BEHIND THE SCENES CALCULATION

[PROGRAMMER: CALCULATE TOTAL TIME IN WEEKS BASED ON 1 MONTH = 4.33 WEEKS & 1 YEAR = 52 WEEKS + WEEKS INDICATED FROM Q530]

L_L_L WEEK(S)

BASE: WAITED 4 WEEKS OR LONGER (Q553/4 WEEKS OR MORE)
Q535 I am going to read you a list of reasons why some people wait before seeing a physician about their symptoms. For each reason, please tell me whether or not it was a reason you waited before seeing a physician. (INTERVIEWER: READ EACH STATEMENT FROM THE LIST BELOW AND AFTER EACH STATEMENT SAY: 'Was it a reason?')

Yes	No	Not Sure (V)	Decline to Answer (V)
1	2	8	9

[RANDOMIZE IF Q900/1]

1. The symptoms were not severe enough
2. You were afraid that your physician would not take you seriously
3. You thought the symptom(s) might go away by themselves
4. You do not like going to a physician
5. You do not like receiving treatments like medications or injections
6. You were concerned about the cost of a physician visit or treatment
7. You were too busy
8. It was difficult to schedule an appointment with the physician
9. You thought you could manage the symptoms yourself
10. You did not know that the symptoms you experienced were a disease that needed medical attention
11. You were not sure how to describe your symptoms to a physician

HARRIS INTERACTIVE

Published previously by: Choy E, et al BMC Health Serv Res 2010, 10:102 [14]

BASE: ALL QUALIFIED RESPONDENTS (Q99/1)

Q545 Before seeing a physician about your symptoms, how did you deal with your symptoms? (INTERVIEWER: READ LIST. ALLOW MULTIPLE RESPONSES.)

[RANDOMIZE IF Q900/1]

1. Took pain reliever(s) you can buy over-the-counter, without a prescription
2. Took other over-the-counter drugs
3. Used traditional, herbal or natural remedies
4. Made some lifestyle changes
5. Exercised
6. Used relaxation techniques
7. Other
8. None (V) [ANCHOR, E]

98. Not sure (V) [ANCHOR, E]
99. Decline to answer (V) [ANCHOR, E]

BASE: ALL QUALIFIED RESPONDENTS (Q99/1)

Q546 Before you were diagnosed, how much did you know about fibromyalgia? Would you say you…?

(INTERVIEWER: READ LIST)

1. You knew a lot about fibromyalgia
2. You knew something about fibromyalgia
3. You knew very little about fibromyalgia
4. You heard the name of the condition only
5. You never heard of the condition

HARRIS INTERACTIVE

Published previously by: Choy E, et al BMC Health Serv Res 2010, 10:102 [14]

SECTION 600: MAIN QUESTIONNAIRE—FIBROMYALGIA DIAGNOSIS/ATTITUDES TOWARD FIBROMYALGIA

Now I am going to ask you some questions about your fibromyalgia diagnosis.

BASE: ALL QUALIFIED RESPONDENTS (Q99/1)
Q600 From the time you first saw a physician about the symptoms you were experiencing, how long did it take before you were diagnosed with fibromyalgia?

(INTERVIEWER: RECORD LENGTH OF TIME AS INDICATED BY RESPONDENT.

- IF RESPONDENT PROVIDES RESPONSE THAT IS LESS THAN ONE WEEK, I.E 5 DAYS, RECORD AS ONE WEEK.)

I_I_I year(s) (Example: 03)

Q601 I_I_I month(s) (Example: 10)

Q602 I_I_I week(s) (Example: 01)
[YEARS, MONTHS, AND WEEKS MANDATORY (DOUBLE DIGIT)]
[RANGE: 00-95]

Q603 97- Diagnosed on first visit to a physician about my symptoms (V) Exclusive
98 – Not Sure (V) (Exclusive)
99 – Decline to Answer (V) (Exclusive)

BASE: ALL QUALIFIED RESPONDENTS (Q99/1)
Q607 BEHIND THE SCENES CALCULATION

[PROGRAMMER: CALCULATE TOTAL TIME IN WEEKS BASED ON 1 MONTH = 4.33 WEEKS & 1 YEAR = 52 WEEKS + WEEKS INDICATED FROM Q530]

L_L_L WEEK(S)

[PN: IF Q600/NE 97 CONTINUE WITH Q605. IF Q600/97 SKIP TO Q610.]

BASE: NOT DIAGNOSED ON FIRST VISIT (Q600/NE 97)
Q605 All together, how many physicians did you see to receive your fibromyalgia diagnosis?

(INTERVIEWER: ENTER 98 FOR NOT SURE, 99 FOR DECLINE TO ANSWER)

I_I_I physicians [RANGE: 1-95; 98, 99]

HARRIS INTERACTIVE

BASE: ALL QUALIFIED RESPONDENTS (Q99/1)

Q610 From the time you first saw a physician about your symptoms and up until now, what type(s) of physicians did you see about your symptoms of fibromyalgia? **(**INTERVIEWER: READ LIST. ALLOW MULTIPLE RESPONSES.)

[RANDOMIZE IF Q900/1]

1. General Practitioner/Family Practitioner
2. Internist/Internal Medicine
3. OBGYN
4. Rheumatologist
5. Neurologist
6. Psychiatrist
7. Pain Specialist
8. Orthopedist
9. Physical Therapist
10. Anesthesiologist
96. Other
98. Not sure (V,) Exclusive
99. Decline to answer (V) Exclusive

BASE: ALL QUALIFIED RESPONDENTS (Q99/1)

Q616 What kind of physician diagnosed you with fibromyalgia? (INTERVIEWER: READ LIST IF NECESSARY. SINGLE RESPONSE.)

[KEEP THE SAME LIST AS Q610 IF Q900/1]

1. General Practitioner/Family Practitioner
2. Internist/Internal Medicine
3. OBGYN
4. Rheumatologist
5. Neurologist
6. Psychiatrist
7. Pain Specialist
8. Orthopedist
9. Physical Therapist
10. Anesthesiologist
96. Other
98. Not sure (V) Exclusive
99. Decline to answer (V) Exclusive

HARRIS INTERACTIVE

Published previously by: Choy E, et al BMC Health Serv Res 2010, 10:102 [14]

BASE: ALL QUALIFIED RESPONDENTS (Q99/1)
Q623 What kind of physician is currently treating your fibromyalgia? (INTERVIEWER: READ LIST IF NECESSARY. MULTIPLE RESPONSE.)

[KEEP THE SAME LIST AS Q610 IF Q900/1]

1. General Practitioner/Family Practitioner
2. Internist/Internal Medicine
3. OBGYN
4. Rheumatologist
5. Neurologist
6. Psychiatrist
7. Pain Specialist
8. Orthopedist
9. Physical Therapist
10. Anesthesiologist
96. Other
98. Not sure (V) Exclusive
99. Decline to answer (V) Exclusive

BASE: ALL QUALIFIED RESPONDENTS (Q99/1)
Q625 Overall, how easy or how difficult was your experience in receiving a fibromyalgia diagnosis? Would you say…? (INTERVIEWER: READ LIST)

1. Very easy
2. Somewhat easy
3. Neither easy nor difficult
4. Somewhat difficult
5. Very difficult
8. Not sure (v)
9. Decline to answer (v)

BASE: ALL QUALIFIED RESPONDENTS (Q99/1)
Q640 Now, I am going to read you several general statements about fibromyalgia. Based on your personal experience being diagnosed and treated for fibromyalgia, please indicate if you strongly agree, somewhat agree, neither agree nor disagree, somewhat disagree or strongly disagree with each of the following statements. (INTERVIEWER: READ LIST. REPEAT SCALE IF NECESSARY.)

Q641

Strongly Agree	Somewhat Agree	Neither Agree Nor Disagree	Somewhat Disagree	Strongly Disagree	Not Sure (V)	Decline to Answer (V)
1	2	3	4	5	8	9

[RANDOMIZE IF Q900/1]
1. Physicians are well trained to diagnose and treat fibromyalgia
2. Physicians are compassionate with their fibromyalgia patients
3. Fibromyalgia patients have had at least one experience where a physician did not take them seriously
4. Physicians need to spend more time with patients to identify fibromyalgia
5. Physicians think that fibromyalgia patients exaggerate their symptoms
6. Fibromyalgia patients find it difficult to communicate their symptoms to physicians
7. Physicians need to focus more on fibromyalgia symptoms
8. Fibromyalgia is a poorly understood condition

HARRIS INTERACTIVE

Published previously by: Choy E, et al BMC Health Serv Res 2010, 10:102 [14]

9. More information and educational materials are needed for fibromyalgia patients to learn more about their disease

BASE: ALL QUALIFIED RESPONDENTS (Q99/1)

Q645 Thinking about all of the physicians you have seen about your fibromyalgia symptoms, overall, how caring do you feel they have been towards you? Would you say…?

1. Extremely caring
2. Very caring
3. Fairly caring
4. Not very caring
5. Not at all caring
8. Not sure (V)
9. Decline to answer (V)

BASE: ALL QUALIFIED RESPONDENTS (Q99/1)

Q650 In a typical month, how often do you visit a physician about your fibromyalgia symptoms? Would you say…?

1. Less than once a month
2. Once a month
3. Twice a month
4. Three times per month or more
8. Not sure (V)
9. Decline to answer (V)

BASE: ALL QUALIFIED RESPONDENTS (Q99/1)

Q655 On average, how much time per visit do you usually spend with your physician? Please provide your answer in minutes.

I_I_I minutes [RANGE: 0-99]

HARRIS INTERACTIVE

Published previously by: Choy E, et al BMC Health Serv Res 2010, 10:102 [14]

SECTION 700: MAIN QUESTIONNAIRE— IMPACT ON QUALITY OF LIFE

BASE: ALL QUALIFIED RESPONDENTS (Q99/1)

Q705 Now I am going to read out a list of some aspects of your life that may have been impacted by fibromyalgia. For each aspect, please tell me if fibromyalgia has had a very strong impact, strong impact, moderate impact, slight impact or no impact?

(INTERVIEWER: READ OUT LIST AND REPEAT SCALE IF NECESSARY.)

Q706

Very Strong Impact	Strong Impact	Moderate Impact	Slight Impact	No Impact	Not Sure (V)	Decline to Answer (V)
1	2	3	4	5	8	9

[RANDOMIZE IF Q900/1]

1. Overall quality of your life [ANCHOR]
2. Your personal relationships
3. Your ability to keep commitments or appointments
4. Your ability to participate in hobbies
5. Your ability to care for family members and children
6. Your sex life
7. Your physical mobility
8. Your overall mood
9. Your concentration or memory
10. Your motivation or drive

BASE: ALL QUALIFIED RESPONDENTS (Q99/1)

Q715 If you had to estimate, how many days of work did you miss over the past 12 months because of your fibromyalgia? (INTERVIEWER: READ OUT LIST.)

1. None – you have not missed any days
2. 1 to 9 days
3. 10 to 20 days
4. 21 to 40 days
5. More than 40 days
6. You have not been employed in the past 12 months
8. Not sure (V)
9. Decline to answer (V)

HARRIS INTERACTIVE

Published previously by: Choy E, et al BMC Health Serv Res 2010, 10:102 [14]

BASE: ALL QUALIFIED RESPONDENTS (Q99/1)

Q726 Please tell me whether each of the following statements applies to you? (INTERVIEWER: READ LIST.)

Q727

Applies to You	Doesn't Apply to You	Not Sure (V)	Decline to Answer (V)
1	2	8	9

[RANDOMIZE IF Q900/1]

1. Due to your fibromyalgia you are not able to work and do not earn an income
2. Due to your fibromyalgia you can only work sometimes and do not earn as much as you used to earn
3. You lost your job due to your fibromyalgia
4. You have spent a lot of money out-of-pocket on medical care due to your fibromyalgia
5. You have been held back in your career due to your fibromyalgia
6. You have paid out-of-pocket for assistance in performing daily activities due to your fibromyalgia
7. Due to your fibromyalgia the quality of your work has deteriorated

BASE: ALL QUALIFIED RESPONDENTS (Q99/1)

Q731 Now I would like to ask you about the level of understanding your family and friends have expressed towards your condition. Would you say your family and friends have been extremely understanding, very understanding, fairly understanding, not very understanding or not at all understanding about the following? (INTERVIEWER: READ OUT LIST. REPEAT SCALE IF NECESSARY.)

Q732

Extremely Understanding	Very Understanding	Fairly Understanding	Not Very Understanding	Not At All Understanding	Not Sure (V)	Decline to Answer (V)
1	2	3	4	5	8	9

[RANDOMIZE IF Q900/1]

1. Your fibromyalgia overall [ANCHOR]
2. You staying home from a family outing due to your fibromyalgia
3. You taking a sick day at work due to your fibromyalgia
4. You needing help with daily chores due to your fibromyalgia
5. You needing to discuss your fibromyalgia with your family and friends

HARRIS INTERACTIVE

Published previously by: Choy E, et al BMC Health Serv Res 2010, 10:102 [14]

SECTION 800: MAIN QUESTIONNAIRE— FIBROMYALGIA TREATMENT

Now I'm going to ask you some questions about how your fibromyalgia is treated.

BASE: ALL QUALIFIED RESPONDENTS (Q99/1)

Q800 What treatments are you currently using to treat your fibromyalgia? (INTERVIEWER READ LIST. ALLOW MULTIPLE RESPONSES.)

[RANDOMIZE IF Q900/1. RANDOMIZE CODES 1-3 TOGETHER, ANCHORING CODE 3]

1. Pain reliever(s) you can buy over-the-counter, without a prescription
2. Pain reliever(s) prescribed by your physician
3. Other drug(s) prescribed by your physician
4. Sleep aids
5. Counseling with a mental health professional
6. Relaxation techniques
7. Biofeedback
8. Lifestyle changes
9. Exercise

96. Other [ANCHOR]
97 None (V) [ANCHOR, E]
98 Not sure (V)[ANCHOR, E]
99 Decline to answer (V) [ANCHOR, E]

[PN: IF Q800/1-9, 96 CONTINUE WITH Q805. IF Q800/97, 98, 99 SKIP TO Q299.]

BASE: CURRENTLY USING AT LEAST ONE OF THE LISTED TREATMENTS (Q800/1-9, 96)

Q805 How satisfied are you with your current overall treatment's ability to relieve your fibromyalgia symptoms? Would you say…? (INTERVIEWER: READ LIST.)

1. Extremely satisfied
2. Very satisfied
3. Fairly satisfied
4. Not very satisfied
5. Not at all satisfied
8. Not sure (V)
9. Decline to answer (V)

HARRIS INTERACTIVE

Published previously by: Choy E, et al BMC Health Serv Res 2010, 10:102 [14]

BASE: QUALIFIED RESPONDENTS WHO MENTIONED AT LEAST ONE LISTED SYMPTOM AND CURRENTLY USE AT LEAST ONE OF THE LISTED TREATMENTS (Q500/1-14,96 AND Q800/1-9, 96)

Q810 Of the fibromyalgia symptoms that you have experienced, which ones, if any, do you feel are not well managed by your current treatment? (INTERVIEWER: READ LIST. ALLOW MULTIPLE RESPONSES.)

[PN: PROGRAMMER: DISPLAY ONLY SYMPTOMS THAT RESPONDENT MENTIONED IN Q500.]

[RANDOMIZE IF Q900/1]

1. Chronic widespread pain
2. Problems sleeping
3. Fatigue
4. Headaches
5. Facial pain
6. Heightened sensitivity to touch
7. Difficulty concentrating
8. Numbness and/or tingling sensations
9. Feelings of anxiety
10. Feelings of depression
11. Joint pain
12. Stiffness
13. Leg cramps
14. Low back pain
96. Other [ANCHOR]
97. None (V) [ANCHOR, E]
98. Not sure (V) [ANCHOR, E]
99. Decline to answer (V) [ANCHOR, E]

HARRIS INTERACTIVE

Published previously by: Choy E, et al BMC Health Serv Res 2010, 10:102 [14]

SECTION 1000: THANK YOU

BASE: QUALIFIED PATIENTS (Q99/1)
Q299 Those are all the questions we have for you. Thank you very much for participating in this survey. Have a great day.

HARRIS INTERACTIVE

Published previously by: Choy E, et al BMC Health Serv Res 2010, 10:102 [14]
