# Supplementary material for: A patient and physician survey of fibromyalgia across Latin America and Europe
Source: BMC Musculoskelet Disord. 2013 Jun 14;14:188. doi: 10.1186/1471-2474-14-188 (PMC3689629; doi:10.1186/1471-2474-14-188)
Supplement: Additional file 2 — Patient questionnaire a. a Published previously by: Choy E, Perrot S, Leon T, Kaplan J, Petersel D, Ginovker A, Kramer E: A patient survey of the impact of fibromyalgia and the journey to diagnosis. BMC Health Serv Res 2010, 10:102 [17]. [file 1471-2474-14-188-S2.pdf]

HARRIS INTERACTIVE
5 Independence Way
Princeton, NJ 08543

Researcher: Anna Ginovker
Email: aginovker@harrisinteractive.com
Phone: 609-919-2638

Kelly Wolfe
Email: kwolfe@harrisinteractive.com
Phone: 424-263-2145

Project Manager: Michi Chan
Email: mchan@harrisinteractive.com
Phone: 585-214-7961

J38889A

Fibromyalgia Study in Latin America - Physician Survey

Estimated Survey Duration: 15 minutes

CLIENT: Pfizer

Information for Online Surveys

Title for initial survey page (Required for online jobs): Health Facts Survey

Wave Number/Version/Languages (if applicable): Mexican Spanish, Brazilian Portuguese, Venezuelan Spanish

HARRIS INTERACTIVE

SECTION 400: PRELOADS/SCREENING QUESTIONS

BASE: ALL RESPONDENTS
Q455 (QV10) COUNTRY

1. MEXICO
2. BRAZIL
3. VENEZUELA

SECTION 410: SCREENER

BASE: ALL RESPONDENTS
Q900 FOR INTERVIEWER USE ONLY (DO NOT READ): Please indicate below how the interview is/was administered.

1 - Administered using online survey program
2 - Administered using pen and paper questionnaire

BASE: ADMINISTERED USING ONLINE SURVEY PROGRAM (Q900/1)
Q405 [IF Q900/1, DISPLAY:] Hello, my name is ______________ from XXXX, an international survey research firm. May I please speak with (INTERVIEWER: INSERT NAME)? (IF NECESSARY: Let me assure you that this is not a sales call, nor will it lead to one, and your answers will be kept strictly confidential.)

1	Yes, speaking	ASK Q407
2	Yes, will connect	ASK Q407
3	Person unavailable	SCHEDULE CALLBACK
4	Person no longer there	TERMINATE (NO LONGER THERE)
5	Wrong number	TERMINATE (WRONG NUMBER)
8	Not sure (V)	TERMINATE (REFUSAL)
9	Decline to answer (V)	TERMINATE (REFUSAL)

BASE: CONTINUING USING ONLINE SURVEY PROGRAM (Q405/1, 2)
Q407 (IF NECESSARY: Hello, I'm _______________ with XXXXX.) We are conducting a research study about pain and would like to include your opinions. If you qualify for and complete this survey, you will be eligible for an honorarium. Would you be willing to participate in this important research study?

(INTERVIEWER, IF NECESSARY: Your responses will be kept strictly confidential and reported in aggregate along with those of other physicians participating in this study.

1	Yes, willing to participate	ASK Q410
2	No, not willing to participate	TERMINATE (REFUSAL)
3	Willing to participate at a later time	SCHEDULE CALLBACK
8	Not sure (V)	TERMINATE (REFUSAL)
9	Decline to answer (V)	TERMINATE (REFUSAL)

HARRIS INTERACTIVE

BASE: WILLING TO PARTICIPATE IN STUDY AND USING ONLINE SURVEY PROGRAM (Q900/1 & Q407/1) OR PEN AND PAPER QUESTIONNAIRE (Q900/2)

Q410 First we would like to ask a few general questions.

What is your primary medical specialty?
(INTERVIEWER: DO NOT READ LIST. SINGLE RESPONSE.)

1	General/Family Practitioner	
2	Internist	
3	Rheumatologist	
4	Neurologist	
5	Psychiatrist	
6	Pain Specialist	SKIP TO Q480
7	Anesthesiologist	ONLY DISPLAY IF Q455/1
96	Other	TERMINATE AND SKIP TO Q480
98	Not sure (V)	TERMINATE AND SKIP TO Q480
99	Decline to answer (V)	TERMINATE AND SKIP TO Q480

IF Q410/1-5,7 CONTINUE WITH Q415, ALL OTHERS SKIP TO Q420.

BASE: RESPONDENTS FROM ONE OF THE LISTED SPECIALTIES EXCEPT PAIN SPECIALISTS (Q410/1-5, 7)

Q415 Do you have a sub-specialty in pain treatment or pain management?

1. Yes
2. No
8. Not sure (V)
9. Decline to answer (V)

BASE: RESPONDENTS FROM ONE OF THE LISTED SPECIALTIES (Q410/1-7)

Q420 Is your practice…?

1. Private
2. Public
3. Both
8. Not sure (V)
9. Decline to answer (V)

BASE: ALL RESPONDENTS

Q480 – INITIAL CLASSIFICATION QUESTION – BEHIND THE SCENES

PROGRAMMER NOTE:

GET CODE 1 IF:

IF Q900/1

- Willing to continue (Q405/1,2) AND
- Willing to participate (Q407/1) AND
- Primary specialty is General/Family Practitioner, Internist, Rheumatologist, Neurologist, Psychiatrist, Pain Specialist or Anesthesiologist (Q410/1-7) AND
  Reside in Mexico, Brazil, Venezuela (Q455/1-3)

IF Q900/2

- Primary specialty is General/Family Practitioner, Internist, Rheumatologist, Neurologist, Psychiatrist, Pain Specialist or Anesthesiologist (Q410/1-7) AND
- Reside in Mexico, Brazil, Venezuela (Q455/1-3)

ALL OTHERS GET CODE 2

1. Qualified
2. Not Qualified

HARRIS INTERACTIVE

SECTION 400: EXPERIENCE/KNOWLEDGE OF FIBROMYALGIA

BASE: ALL QUALIFIED RESPONDENTS (Q99/1)
Q435 For how many years have you been in practice?
(INTERVIEWER, ENTER '98' FOR NOT SURE OR '99' FOR DECLINE TO ANSWER)

[RANGE: 1-65, 98, 99]
|__|__| year(s)

BASE: ALL QUALIFIED RESPONDENTS (Q99/1)
Q440 Approximately how many patients per month do you see yourself in your practice overall? *Your best estimate is fine.*

(INTERVIEWER: Enter 9998 for Not Sure, and 9999 for Decline to Answer.)

[RANGE 1-9999]
|_|_|_|_

BASE: ALL QUALIFIED RESPONDENTS (Q99/1)
Q445 Are you currently seeing or have you seen patients over the past 2 years diagnosed with the following conditions?

(INTERVIEWER: READ LIST. ALLOW MULTIPLE RESPONSES.)
[RANDOMIZE IFQ900/1]

1. Migraines
2. Arthritis
3. Chronic fatigue syndrome
4. Depression
5. Irritable bowel syndrome
6. Lyme disease
7. Fibromyalgia
8.. Lupus
9. Multiple Sclerosis
97. None of the above [ANCHOR, E]
98. Not sure (ANCHOR, V, E)
99. Decline to answer (ANCHOR, V, E)

IF Q445/7 CONTINUE WITH Q460. IF Q445/NE7, SKIP TO Q470.

BASE: CURRENTLY SEEING/HAVE SEEN PATIENTS DIAGNOSED WITH FIBROMYALGIA (Q445/7)
Q460 Approximately, how many patients diagnosed with fibromyalgia have you seen in the past 2 years including those you are currently seeing?

(INTERVIEWER: Enter '9998' for Not Sure, '9999' for Decline to Answer.)

[RANGE 1-9999]
|_|_|_|

HARRIS INTERACTIVE

BASE: CURRENTLY SEEING/HAVE SEEN PATIENTS DIAGNOSED WITH FIBROMYALGIA (Q445/7)

Q465 What percentage of the fibromyalgia patients that you have seen in the past 2 years, including those you are currently seeing, are women?

(INTERVIEWER: Enter '998' for Not Sure, '999' for Decline to Answer.)

[RANGE 0-100, 998, 999]
|_|_|_| % women

BASE: CURRENTLY SEEING/HAVE SEEN PATIENTS DIAGNOSED WITH FIBROMYALGIA (Q445/7)

Q468 Now, please think about all the patients who have come to you over the past 2 years **either directly or by referral from another physician presenting with symptoms of fibromyalgia, but not yet diagnosed with fibromyalgia.** Would you say that the number of those patients has increased, decreased or stayed the same over the past 2 years?

1. Increased
2. Decreased
3. Stayed the same
8. Not sure (V)
9. Decline to answer (V)

BASE: CURRENTLY SEEING/HAVE SEEN PATIENTS DIAGNOSED WITH FIBROMYALGIA (Q445/7)

Q469 Over the past two years, has the number of patients in your practice actually **diagnosed with fibromyalgia either by you or by another physician** increased, decreased or stayed the same?

1 Increased
2 Decreased
3 Stayed the same
8 Not sure (V)
9 Decline to answer (V)

BASE: ALL QUALIFIED RESPONDENTS (Q99/1)

Q470 Now, I would like to ask you some general questions about fibromyalgia. How would you rate your level of knowledge about fibromyalgia? Would you say you are…?

1. Very knowledgeable
2. Knowledgeable
3. Not very knowledgeable
4. Not at all knowledgeable
8. Not sure (V)
9. Decline to answer (V)

IF Q445/7 CONTINUE WITH Q500. All OTHERS SKIP TO Q515.

HARRIS INTERACTIVE

SECTION 500: DIAGNOSIS OF FIBROMYALGIA

BASE: CURRENTLY SEEING/SEEN PATIENTS WITH FIBROMYALGIA (Q445/7)

Q500 Now, please think of all the patients you have seen with fibromyalgia in the past 2 years, including those you are currently seeing. Which of the following statements describe the ways you handle diagnosis and treatment of your fibromyalgia patients? *(INTERVIEWER: READ EACH ITEM. MARK ALL THAT APPLY)*

[MULTIPLE RESPONSE] [RANDOMIZE IFQ900/1]

1. You diagnose and then refer your fibromyalgia patients to another physician for treatment
2. You diagnose and also treat your fibromyalgia patients
3. You treat fibromyalgia patients who were diagnosed by another physician and then referred to you for treatment
8. Not sure (V)
9. Decline to answer (V)

IF Q500/3 CONTINUE WITH Q505. IF Q500/1 SKIP TO Q510. IF Q500/2 SKIP TO Q515.

BASE: HAVE PATIENTS WHO WERE REFERRED TO THEM (Q500/3)

Q505 Thinking about the patients who were diagnosed by another physician and then referred to you for treatment over the past 2 years, which physician specialties commonly refer fibromyalgia patients to you? *(INTERVIEWER: READ LIST. ALLOW MULTIPLE RESPONSES. IF RESPONDENT MENTIONS A SPECIALTY NOT LISTED, CODE UNDER 'OTHER'.)*
[RANDOMIZE IFQ900/1]

1 General / Family Practitioner
2 Internists
3 Rheumatologists
4 Neurologists
5 Psychiatrists
6 Pain Specialists
7 OBGYN
8 Orthopedists
9 Anesthesiologists
10 Physical Therapists
96 Other
98 Not sure (V), Exclusive
99 Decline to answer (V), Exclusive

BASE: HAVE PATIENTS WHO HAVE DIAGNOSED AND THEN REFERRED (Q500/1)

Q510 You have indicated that over the past 2 years you have referred fibromyalgia patients to another physician for treatment. Why did you refer those patients to another physician? Would you say…? *(INTERVIEWER: READ LIST. MARK ALL THAT APPLY.)*

[MULTIPLE RESPONSE] [RANDOMIZE IFQ900/1]

1. You were not familiar with treatments appropriate for patients with fibromyalgia
2. You had limited experience with fibromyalgia
3. You did not have time to treat fibromyalgia patients over the long term
8. Not sure (V), Exclusive
9. Decline to answer (V), Exclusive

HARRIS INTERACTIVE

BASE: ALL QUALIFIED RESPONDENTS (Q99/1)

Q515 From your experience and/or what you may have read or heard, how easy or how difficult is it to diagnose fibromyalgia? Would you say…? *(INTERVIEWER: READ LIST)*

1. Very easy
2. Somewhat easy
3. Neither easy nor difficult
4. Somewhat difficult
5. Very difficult
8. Not sure (V)
9. Decline to answer (V)

BASE: ALL QUALIFIED RESPONDENTS (Q99/1)

Q520 I am going to read you several statements some physicians make about diagnosing fibromyalgia. From your experience and/or what you may have read or heard, please tell me if you strongly agree, somewhat agree, neither agree nor disagree, somewhat disagree, or strongly disagree with each of the following statements.

Q521

Strongly Agree	Somewhat Agree	Neither Agree Nor Disagree	Somewhat Disagree	Strongly Disagree	Not Sure (V)	Decline to Answer (V)
1	2	3	4	5	8	9

(INTERVIEWER: READ LIST. REPEAT SCALE AS NECESSARY.) [RANDOMIZE IFQ900/1]

1. It is difficult for patients to communicate symptoms of fibromyalgia to a physician
2. Patients tend to delay seeking medical help even when symptoms of fibromyalgia appear
3. Physicians are not always comfortable diagnosing fibromyalgia
4. The symptoms of fibromyalgia can often be difficult to discriminate from the symptoms of other conditions
5. Physicians need to spend more time with patients to identify fibromyalgia
6. Patients often see multiple physicians before receiving fibromyalgia diagnosis
7. Fibromyalgia is not well understood among many physicians
8. Fibromyalgia is not well understood among patients

BASE: ALL QUALIFIED RESPONDENTS (Q99/1)

Q525 From your experience and/or what you may have read or heard, how often do you think fibromyalgia is misdiagnosed? Would you say…?

1. Almost always
2. Often
3. Sometimes
4. Rarely
5. Never
8. Not sure (V)
9. Decline to answer (V)

HARRIS INTERACTIVE

BASE: ALL QUALIFIED RESPONDENTS (Q99/1)

Q530 And how confident are you personally about each of the following? Would you say you are very confident, confident, not very confident, or not at all confident?

Q531

Very Confident	Confident	Not Very Confident	Not At All Confident	Not Sure (V)	Decline to Answer (V)
1	2	3	4	8	9

(INTERVIEWER: READ LIST. REPEAT SCALE AS NECESSARY.)

[DO NOT RANDOMIZE]

1. Recognizing the symptoms of fibromyalgia
2. Differentiating fibromyalgia from conditions with similar symptoms
3. Conducting tender points examination
4. Developing treatment plans for patients diagnosed with fibromyalgia
5. Managing fibromyalgia patients over the long-term

BASE: ALL QUALIFIED RESPONDENTS (Q99/1)

Q535 Which of the following best describes the level of fibromyalgia training that you received? *(INTERVIEWER: READ LIST. SINGLE RESPONSE)*

1 You received extensive fibromyalgia training
2 You received some fibromyalgia training
3 You received very little fibromyalgia training
4 You did not receive any fibromyalgia training at all
8 Not sure (V)
9 Decline to answer (V)

BASE: ALL QUALIFIED RESPONDENTS (Q99/1)

Q540 Are you aware of the fibromyalgia diagnostic criteria published by the American College of Rheumatology (ACR) in 1990?

1. Yes
2. No
8. Not sure (V)
9. Decline to answer (V)

IF Q540/1 AND Q445/7 CONTINUE WITH Q545. All OTHERS SKIP TO Q600.

HARRIS INTERACTIVE

BASE: CURRENTLY SEEING/HAVE SEEN PATIENTS WITH FIBROMYALGIA AND ARE AWARE OF CRITERIA (Q445/7 AND Q540/1)

Q545 Do you use the American College of Rheumatology (ACR) diagnostic criteria to identify fibromyalgia in your clinical practice?

1. Yes
2. No
8. Not sure (V)
9. Decline to answer (v)

IF Q545/2, 8. 9 CONTINUE WITH Q550. All OTHERS SKIP TO Q600.

BASE: DO NOT USE CRITERIA IN PRACTICE (Q545/2, 8, 9)

Q550 I'm going to read you several statements that describe the reasons why you may not use the ACR criteria to identify fibromyalgia. For each statement, please tell me if it is a reason why you would not use the ACR criteria. [*INTERVIEWER: READ EACH STATEMENT FROM THE LIST AND AFTER EACH STATEMENT SAY: '*Is it a reason why you would not use the ACR criteria*?']*

[RANDOMIZE IFQ900/1]

Q551

Yes	No	Not Sure (V)	Decline to Answer (V)
1	2	8	9

1. You do not feel that the usage of the criteria yields an accurate diagnosis
2. You do not have enough time to use ACR criteria
3. You depend more on your own clinical judgment than diagnostic criteria
4. You do not have sufficient experience using ACR criteria

IF Q445/NE7 CONTINUE WITH Q600. IF Q445/7 SKIP TO Q605.

HARRIS INTERACTIVE

SECTION 600: SYMPTOMS OF FIBROMYALGIA

BASE: CURRENTLY NOT SEEING / HAVE NOT SEEN PATIENTS WITH FIBROMYALGIA (Q445/NE7)

Q600 Next, I am going to read you some symptoms of fibromyalgia. Based on what you may have read or heard about fibromyalgia, please tell me how typical each one is as a symptom of the disease – most typical, very typical, typical, not too typical, or not at all typical. (*INTERVIEWER: READ LIST AND REPEAT SCALE IF NECESSARY.)*

Q601

Most Typical	Very Typical	Typical	Not Too Typical	Not At All Typical	Not Sure(V)	Decline to Answer (V)
1	2	3	4	5	8	9

[RANDOMIZE IFQ900/1]

1. Chronic widespread pain
2. Problems sleeping
3. Fatigue
4. Headaches
5. Facial pain
6. Heightened sensitivity to touch
7. Difficulty concentrating
8. Numbness and/or tingling sensations
9. Feelings of anxiety
10. Feelings of depression
11. Joint pain
12. Stiffness
13. Leg cramps
14. Low back pain

IF Q445/NE7 SKIP TO Q700. IF Q445/7 ASK Q605.

BASE: CURRENTLY SEEING / HAVE SEEN PATIENTS WITH FIBROMYALGIA (Q445/7)

Q605 Next, I am going to read you a list of symptoms of fibromyalgia. What is the one <u>most typical</u> symptom you look for when diagnosing this condition? *(INTERVIEWER: READ LIST. SINGLE RESPONSE.)*

[RANDOMIZE IFQ900/1]

1. Chronic widespread pain
2. Problems sleeping
3. Fatigue
4. Headaches
5. Facial pain
6. Heightened sensitivity to touch
7. Difficulty concentrating
8. Numbness and/or tingling sensations
9. Feelings of anxiety
10. Feelings of depression
11. Joint pain
12. Stiffness
13. Leg cramps
14. Low back pain

97. None of the above (V, E) [ANCHOR]
98. Not sure (V, E) [ANCHOR]
99. Decline to answer (V, E) [ANCHOR

HARRIS INTERACTIVE

BASE: CURRENTLY SEEING / HAVE SEEN PATIENTS WITH FIBROMYALGIA (Q445/7)

Q610 Again, thinking about the symptoms of fibromyalgia, how disruptive you think each of them is to the overall quality of the patients' lives? Would you say it is not at all disruptive, not very disruptive, fairly disruptive, very disruptive, or extremely disruptive? (*INTERVIEWER: READ LIST AND REPEAT SCALE IF NECESSARY)*

Q611

Not At All Disruptive	Not very Disruptive	Fairly Disruptive	Very Disruptive	Extremely Disruptive	Not Sure (V)	Decline to Answer (V)
1	2	3	4	5	8	9

[RANDOMIZE IFQ900/1 AND KEEP SAME ORDER AS Q605]

1. Chronic widespread pain
2. Problems sleeping
3. Fatigue
4. Headaches
5. Facial pain
6. Heightened sensitivity to touch
7. Difficulty concentrating
8. Numbness and/or tingling sensations
9. Feelings of anxiety
10. Feelings of depression
11. Joint pain
12. Stiffness
13. Leg cramps
14. Low back pain

HARRIS INTERACTIVE

SECTION 700: ATTITUDES AND BELIEFS ABOUT FIBROMYALGIA

BASE: ALL QUALIFIED RESPONDENTS (Q99/1)
Q700 Now, I will read you several general statements about fibromyalgia. Based on your experience and/or what you may have read or heard, please indicate whether you strongly agree, somewhat agree, neither agree nor disagree, somewhat disagree, or strongly disagree with each of the following statements? (*INTERVIEWER READ LIST. REPEAT SCALE AS NECESSARY)*

[RANDOMIZE IFQ900/1]

Q701

Strongly Agree	Somewhat Agree	Neither Agree Nor Disagree	Somewhat Disagree	Strongly Disagree	Not Sure (V)	Decline to Answer (V)
1	2	3	4	5	8	9

1. Physicians are well trained to diagnose and treat fibromyalgia
2. Physicians are compassionate with their fibromyalgia patients
3. Most fibromyalgia patients have had at least one experience of a physician not taking them seriously
4. Fibromyalgia patients exaggerate their symptoms
5. Physicians need to focus more on fibromyalgia symptoms
6. Patients are often unaware of fibromyalgia until they are actually diagnosed with this condition
7. More information and educational materials are needed for fibromyalgia patients to learn more about their disease
8. More information and educational materials are needed for physicians to learn more about fibromyalgia
9. Fibromyalgia is a debilitating chronic pain condition

IFQ445/7 CONTINUE WITH Q705. ALL OTHERS SKIP TO Q800.

HARRIS INTERACTIVE

BASE: CURRENTLY SEEING / HAVE SEEN PATIENTS WITH FIBROMYALGIA (Q445/7)

Q705 And now I would like to ask you about the impact of fibromyalgia on your patients' lives. I am going to read you a list of some aspects of your patients' lives that may have been impacted by fibromyalgia. Please tell me if you think fibromyalgia has had a very strong impact, strong impact, moderate impact, slight impact or no impact on each of the following aspects.

(INTERVIEWER: READ OUT LIST AND REPEAT SCALE IF NECESSARY.)

[RANDOMIZE IFQ900/1]

Q706

Very Strong Impact	Strong Impact	Moderate Impact	Slight Impact	No Impact	Not Sure (V)	Decline to Answer (V)
1	2	3	4	5	8	9

1. Overall quality of patients lives [ANCHOR]
2. Their personal relationships
3. Their ability to keep commitments or appointments
4. Their ability to participate in hobbies
5. Their ability to care for family members and children
6. Their sex life
7. Their physical mobility
8. Their overall mood
9. Their concentration or memory
10. Their motivation or drive
11. Their ability to work
12. Their finances

HARRIS INTERACTIVE

SECTION 800: TREATING FIBROMYALGIA

BASE: ALL QUALIFIED RESPONDENTS (Q99/1)

Q800 From your experience and/or what you may have read or heard, how easy or how difficult is it to treat fibromyalgia? Would you say…? *(INTERVIEWER: READ LIST)*

1. Very easy
2. Somewhat easy
3. Neither easy nor difficult
4. Somewhat difficult
5. Very difficult
8. Not sure (V)
9. Decline to answer (V)

BASE: ALL QUALIFIED RESPONDENTS (Q99/1)

Q805 Now, I am going to read you several statements about treating fibromyalgia. From your experience and/or what you may have read or heard about treating fibromyalgia, please indicate whether you strongly agree, somewhat agree, neither agree nor disagree, somewhat disagree, or strongly disagree with each of the following statements? (*INTERVIEWER READ LIST. REPEAT SCALE AS NECESSARY.)*

[RANDOMIZE IFQ900/1]

Q806

Strongly Agree	Somewhat Agree	Neither Agree Nor Disagree	Somewhat Disagree	Strongly Disagree	Not Sure (V)	Decline to Answer (V)
1	2	3	4	5	8	9

1. Treating fibromyalgia can be very time consuming for physicians
2. Treating fibromyalgia requires frequent patient visits
3. It is difficult to gauge progress in treating fibromyalgia
4. With so many symptoms of fibromyalgia, many different medications are needed to treat it
5. Simpler guidelines are needed to improve the treatment of fibromyalgia
6. Better medications/more effective medications are needed for treating fibromyalgia
7. More educational programs should be available to help physicians learn more about treating fibromyalgia

IF Q445/7 CONTINUE. ALL OTHERS SKIP TO Q825.

BASE: CURRENTLY SEEING OR HAVE SEEN FIBROMYALGIA PATIENTS (Q445/7)

Q810 Overall, how would you rate the currently available treatments for fibromyalgia? Would you say…? *(INTERVIEWER: READ LIST)*

1. Excellent
2. Very good
3. Good
4. Fair
5. Poor
8. Not sure (V)
9. Decline to answer (V)

HARRIS INTERACTIVE

BASE: CURRENTLY SEEING OR HAVE SEEN FIBROMYALGIA PATIENTS (Q445/7)
Q815 When treating patients for fibromyalgia, what type of treatments do you use most frequently? Please select those treatments which you use most frequently.

[RANDOMIZE IFQ900/1]

1. Analgesics
2. Antidepressants
3. Antiepileptics
4. Anti-inflammatories/NSAIDs
5. Anxiety treatments/benzodiazepines
6. Muscle Relaxants
7. Neuromodulators
8. Opiods
9. Vitamin B12
97. None of the above (V, E) [ANCHOR]
98. Not sure (V, E) [ANCHOR]
99. Decline to answer (V, E) [ANCHOR]

BASE: CURRENTLY SEEING OR HAVE SEEN FIBROMYALGIA PATIENTS (Q445/7)
Q820 Overall, what symptom(s) of fibromyalgia, if any, do you feel are not adequately treated by current treatment options? (*INTERVIEWER: READ LIST. ALLOW MULTIPLE RESPONSES.)*

[RANDOMIZE IFQ900/1]

1. Chronic widespread pain
2. Problems sleeping
3. Fatigue
4. Headaches
5. Facial pain
6. Heightened sensitivity to touch
7. Difficulty concentrating
8. Numbness and/or tingling sensations
9. Feelings of anxiety
10. Feelings of depression
11. Joint pain
12. Stiffness
13. Leg cramps
14. Low back pain
97. None of the above (V, E) [ANCHOR]
98. Not sure (V, E) [ANCHOR]
99. Decline to answer (V, E) [ANCHOR]

BASE: ALL QUALIFIED RESPONDENTS (Q99/1)
Q825 Do you have specific fibromyalgia treatment guidelines in your country?

1. Yes
2. No
8. Not sure (V)
9. Decline to answer (V)

IF Q825/1 AND Q445/7 CONTINUE WITH Q830. All OTHERS SKIP TO Q835.

HARRIS INTERACTIVE

BASE: CURRENTLY SEEING OR HAVE SEEN FIBROMYALGIA PATIENTS AND HAVE SPECIFIC TREATMENT GUIDELINES (Q445/7 AND Q825/1)

Q830 Do you use the fibromyalgia treatment guidelines in your practice?

1. Yes
2. No
8. Not sure (V)
9. Decline to answer (V)

BASE: ALL QUALIFIED RESPONDENTS (Q99/1)

Q835 To your knowledge, are there any medications specifically approved for the treatment of fibromyalgia in your country?

1. Yes
2. No
8. Not sure (V)
9. Decline to answer (V)

HARRIS INTERACTIVE

SECTION 900: INCLUSION OF FIBROMYALGIA INTO INSTITUTION FORMULARY

BASE: ALL QUALIFIED RESPONDENTS (Q99/1)

Q900 To the best of your knowledge, is fibromyalgia coded in the official index of conditions in your country's healthcare system or institution formulary?

1. Yes
2. No
8. Not sure (V)
9. Decline to answer (V)

IF Q900/2,8,9 CONTINUE WITH Q905. IF Q900/1 SKIP TO Q910.

BASE: RESPONDENTS WHO STATE THAT FIBROMYALGIA IS NOT INCLUDED IN THE LIST (Q900/2,8,9)

Q905 How important do you think coding fibromyalgia in the official index of conditions is for improved fibromyalgia patient care? Would you say…? (INTERVIEWER: READ LIST)

1. Absolutely essential
2. Very important
3. Fairly important
4. Not very important
5. Not at all important
8. Not sure (V)
9. Decline to answer (V)

BASE: ALL QUALIFIED RESPONDENTS (Q99/1)

Q910 Now, I'm going to read several statements that describe the benefits of coding fibromyalgia in the official index of conditions used in your country's healthcare system or institution formulary. Please indicate whether you strongly agree, somewhat agree, neither agree nor disagree, somewhat disagree, or strongly disagree with each of the following statements. (*INTERVIEWER READ LIST. REPEAT SCALE AS NECESSARY.)*

Q911

Strongly Agree	Somewhat Agree	Neither Agree Nor Disagree	Somewhat Disagree	Strongly Disagree	Not Sure (V)	Decline to Answer (V)
1	2	3	4	5	8	9

[RANDOMIZE IFQ900/1]

1. Coding fibromyalgia in the official index of conditions improves access to treatment
2. Coding fibromyalgia in the official index of conditions increases awareness of the disease
3. Coding fibromyalgia in the official index of conditions reduces time to accurately diagnose patients

HARRIS INTERACTIVE

SECTION 1000: THANK YOU

BASE: ALL RESPONDENTS (Q99/1)
Q1005 These are all the questions that we have. Thank you very much for your participation in this important research. We greatly appreciate your time and input. Have a great day.

HARRIS INTERACTIVE
